# New approaches to surveillance and control of emerging foodborne infectious diseases.

**DOI:** 10.3201/eid0403.980329

**Published:** 1998

**Authors:** R V Tauxe

**Affiliations:** Centers for Disease Control and Prevention, Atlanta, Georgia, USA.


### New Approaches to Surveillance and Control of Emerging Foodborne Infectious Diseases

**Robert V. Tauxe**
Centers for Disease Control and Prevention, Atlanta, Georgia, USA

Each year in the United States, foodborne diseases affect millions of persons, who become ill after exposure to any of a growing spectrum of identified agents and toxins. Typhoid fever and other foodborne diseases common a century ago have been controlled by measures that prevent contamination of food and water with human sewage and by technologies (such as milk pasteurization) that eliminate any remaining pathogens. Many recently identified foodborne diseases are caused by contamination with animal feces and can be prevented by measures that reduce contamination and eliminate residual pathogens. In the future, growing attention will need to be directed to the safety of the food and water the animals themselves consume.

New foodborne diseases emerge for many reasons, including changes in the pathogens themselves, increasingly centralized and concentrated food production, globalization of the food supply, and increases in populations at higher risk. Better surveillance and investigation now detect outbreaks that a few years ago would have been missed. The continuing challenges are to identify new pathogens as they emerge, understand how foodborne pathogens contaminate food and cause illness, and define and implement the best prevention strategies.

Many efforts are now under way to improve food safety in the United States. In 1997, the National Food Safety Initiative outlined an interagency effort to enhance foodborne disease surveillance, research, and prevention. The Centers for Disease Control and Prevention (CDC) and state and local health departments have begun to implement improved surveillance strategies, including additional resources for basic surveillance and investigation, an active surveillance network called FoodNet, surveillance for antimicrobial resistance, and a network for molecular subtyping called PulseNet. Basic research at the National Institutes of Health (NIH) is clarifying virulence mechanisms and developing prevention tools. Dennis Lang, National Institute of Allergy and Infectious Diseases, emphasized that NIH-supported investigators who study the organisms responsible for foodborne illness represent a national resource that can be used to address food safety questions more effectively. New approaches to prevention are now being implemented by the food regulatory agencies, and more approaches, including irradiation, have been approved for industry use.

Barbara Herwaldt, CDC, reported that *Cyclospora cayetanensis* is an archetypical emerging foodborne pathogen. This recently described parasitic pathogen sprang to national attention in nationwide outbreaks in 1996, which were traced to raspberries imported from Guatemala. Outbreaks recurred in 1997, leading to a suspension of importation, despite efforts of the Guatemalan raspberry industry to reduce potential contamination. With improved surveillance, other outbreaks were detected, investigated, and traced to mesclun lettuce and basil. CDC investigation has now documented *C. cayetanensis* as a common cause of springtime diarrhea among children in Guatemala. Critical gaps in our understanding of the biology and epidemiology of this parasite, particularly in the raspberry farm environment, need to be closed before effective control measures can be developed.

B. Swaminathan, CDC, described a new subtyping strategy for public health surveillance of *Escherichia coli* O157:H7 that will become available electronically later this year. This strategy depends on standardized molecular fingerprinting in public health and food regulatory agency laboratories by pulsed-field gel electrophoresis (PFGE). With standardized methods and equipment, excellent interlaboratory comparability of DNA fingerprint patterns has been achieved. Twenty-four states, the U.S. Department of Agriculture, and the Food and Drug Administration are now equipped to use CDC's PFGE method for *E. coli* O157:H7. These laboratories are being linked to form a collaborative network for molecular subtyping, PulseNet, which will permit rapid comparison of identified PFGE profiles with the national database at CDC. Efforts are also under way to apply the same strategy to other foodborne pathogens. In 1997, PFGE results were already critical to epidemiologic investigations of several outbreaks of *E. coli* O157:H7 infections. These included a Colorado outbreak traced to ground beef and a multistate outbreak related to alfalfa sprouts.

Alison O'Brien, Uniformed Services University of the Health Sciences, described a new approach to prevention, based on an attachment protein present in enteropathogenic *E. coli* as well as *E. coli* O157:H7. This protein, intimin, permits the bacteria to attach to mucosal cells and produce a characteristic pathologic change. In a calf model, *E. coli* O157:H7 can cause diarrhea and this change; the change does not occur if the *E. coli* lack the gene for intimin. Intimin is highly antigenic and acid stable, and antibodies raised to it block adherence in vitro. The intimin gene has been introduced into plants, where it is produced in the leaves. This means that an antitransmission vaccine based on intimin can be produced cheaply in plants and be given to calves. The vaccine could even be fed to animals if intimin were produced in fodder plants. In the future, we may be able to prevent *E. coli* O157:H7 in humans by vaccinating the bovine reservoir.

Henrik Wegener, Danish Zoonosis Center, described the emergence of vancomycin-resistant enterococci (VRE) in northern Europe, linking it to the use of a related glycopeptide antibiotic, avoparsin, in food animals. VRE were common in poultry flocks and swine herds exposed to this antibiotic, and 5% of healthy carnivorous humans were carriers of VRE. Sequencing the resistance gene showed that one genotype was present in poultry, a second was present in swine, and both were present in humans. Thus, VRE are unlikely to have spread from animals to humans rather than vice versa. After avoparsin was withdrawn in Denmark in 1995, the prevalence of VRE in chickens dropped; the European Union banned the agent in 1997. In some countries, amplification of VRE in hospitals where vancomycin use is frequent may follow introduction of resistance strains from food sources. Other antibiotics being developed for human use (e.g., streptogramins) have analogues used in agriculture for years, to which resistance may already have emerged. Integrated resistance surveillance systems, data on antibiotic use in humans and in agriculture, and prudent agricultural use policies are critical to managing the growing challenge of antibiotic resistance related to foods and food animals.

